# Relationship between core muscle strength and dynamic balance among hospital staff

**DOI:** 10.1186/s43161-022-00082-y

**Published:** 2022-06-15

**Authors:** Nawaf Almutairi, Ahmad Alanazi, Mohammed Seyam, Faizan Zaffar Kashoo, Danah Alyahya, Radhakrishnan Unnikrishnan

**Affiliations:** 1 Department of Physical Therapy, Rabigh General Hospital, Rabigh, Saudi Arabia; 2 Department of Physical Therapy and Health Rehabilitation, College of Applied Medical Sciences, Majmaah University, Majmaah, Province Riyadh Saudi Arabia

**Keywords:** Core muscles strength, Dynamic balance, Hospital staff

## Abstract

**Background:**

Healthcare workers are at the risk of developing weakness in core muscles and balance disturbance due to stress at the workplace. The purpose of this study was to examine the relationship between core muscle strength measured with a plank test and dynamic balance assessed with the modified Star Excursion Balance Test (MSEBT) among hospital staff. A convenience sample of 27 healthy male employees at Rabigh General Hospital participated in the study; participants performed MSEBT and plank tests in the gym of the physical therapy department at the hospital.

**Results:**

The mean age of the 27 participants was 32.19, standard deviation (SD) 4.16 years; mean height was 171.15, SD 6.39 cm; mean weight was 72.37, SD 11 kg; and body mass index was 24.73, SD 3.62 kg/m^2^. Pearson’s correlation coefficient showed a positive significant correlation between scores on the plank test with leg reach scores on MSEBT. The data showed a highest correlation between scores on plank test with dominant anterior leg reach scores on MSEBT (*r* = 0.446, *p* = 0.010), and lowest with non-dominant anterior leg reach scores on MSEBT (*r* = 0.335, *p* = 0.044).

**Conclusion:**

Weak to moderate positive significant correlation between the plank test of isometric core muscle strength and both the right and dominant of the anterior, posteromedial, and composite score on the MSEBT of the lower limb and significantly with non-dominant anterior reach. There was no significant difference between the administrative and health practitioner on the plank test or MSEBT.

## Background

Healthcare workers (HCWs) play an important role in patient care. Hospital workers and nurses provide essential services to the patients in public health clinics. The healthcare field is also one of the fast-growing job markets globally due to the increasing number of aged population and diseases. One of the common musculoskeletal disorders reported in the scientific literature was the weak core muscle [1–3]. HCWs are at the risk of developing weakness in the core muscles and musculoskeletal disorders due to the busy work schedule and occupational stress [4, 5]. Reports from the Centers for Disease Control and Prevention (CDC) in the USA have estimated 18 million healthcare staff annually are at a greater risk of developing suffering musculoskeletal disorders during their employment [6].

There is a group of unique core muscles (pelvic floor muscles, transverses abdominis, multifidus, internal and external oblique’s, rectus abdominis, erector spinae, and diaphragm) work independently to contribute to the overall stability of the spine. These core muscles of the trunk were found to be more important for spine stabilization, especially the transverses abdominis (TrA). Studies have reported that the delay in the activation of core muscles is associated with chronic low back pain (CLBP) [7]. The studies on core muscle function are important in understanding neuromuscular risk factors associated with CLBP [8]. According to Kibler and colleagues, the muscles of the core form a rigid cylinder with a higher moment against body disorder and produce a strong basis for mobility. The abdominal muscles, which include the internal and external obliques, transverse abdominis, and rectus abdominis, all contract to produce spinal stability and thus a greater foundation of strength in the movement of the lower extremity [9]. The transverses abdominis has also been shown to be significant in lumbar spine stabilization. When it contracts, it raises intra-abdominal pressure and tightens the thoracolumbar fascia [9] Core muscle contraction occurs before the start of limb movement, providing the limbs with a strong foundation for motion and muscle activation [10]. The oblique abdominals and rectus abdominis are excited in limb movement-specific patterns, providing postural protection before limb movements [9]. Retraining the core muscles is reported to alleviate pain and improve static and dynamic spine balance [11]. Exercises are designed to improve endurance and initiation (onset of contraction) of core muscles more than mere strength. Exercises such as bird and dog and cat and camel along with tummy tucks in quadruped position are reported to improve patients with low back pain and athletes as a preventative strategy [12].

The ability of the individual to maintain the center of gravity (COG) within the base of support during movement is known as dynamic balance. Dynamic equilibrium is important for everyday activities such as stair climbing, hiking, and walking. It's also a major factor in lower-limb injuries [13]. Balance, in general, refers to the maintenance to hold the center of gravity inside the base of support which may be dynamic or static. Static balance is characterized as holding the body inside the base of support when in static equilibrium. Dynamic balance is more difficult to achieve because it requires the ability to preserve equilibrium while transitioning from a dynamic to a static state. Both dynamic and static equilibrium requires an effective visual system. Inputs from the integrated visual, proprioceptive, and vestibular systems are combined to provide an efferent response to body control within the base of support [14].

There is scarcity of research published related to the association of core muscle strength and balance disorders in healthcare providers. A purpose was to examine the relationship between core muscle strength and dynamic balance among hospital staff. It was hypothesized that there would be no significant difference between administrative and health practitioners in dynamic balance and core muscle strength

## Methods

### Participants

The participants were eligible to participate in our study if they were allied health care workers or hospital administrative staff employed at Rabigh General Hospital in Rabigh city, Saudi Arabia. Individuals with musculoskeletal injury and neurological, vestibular, or balance disorders were excluded. Twenty-seven healthy male employees through convenience sampling method consented to participate with mean (M) age of 32.19 and standard deviation (SD) of ± 4.16 years; height: M=171.15, SD ± 6.39 cm; weight: M=72.37, SD ± 11 kg; and BMI: M=24.73, SD± 3.62 kg/m^2^. Out of the 27 participants in our study, 14 were physiotherapists and the remaining were administrative staff.

### Measurements

#### Modified Star Excursion Balance Test (MSEBT)

The MSEBT is a concise version of the Star Excursion Balance Test (SEBT) and is used to assess postural control, strength, and proprioception [15]. The MSEBT was administered by instructing the person to stand on one leg while the other leg reaches in three different directions (anterior, posterolateral, and posteromedial). A composite score was then determined by adding the distances achieved in the three reach directions. The MSEBT is a reliable and valid tool for quantitative balance assessment [16]. The participants were instructed to extend one leg maximally in three directions (ANT, PM, and PL) without changing the position of their stance leg. The participants were instructed to place hands on their hips while performing the test. The test could be used to evaluate athletic capacity, but it could also be used to check for deficiencies in complex postural control caused by musculoskeletal injury [15]. All reach distances were normalized as a percentage of the stance limb length (LL) using the formula [% = (excursion distance/LL) × 100]. A composite score, which is an average of all three reach distances, [Comp= ((ANT+PM+PL)/(3 × LL)) × 100] was also calculated for each limb. The absolute difference in the anterior reach direction distance (centimeters) between limbs was calculated to assess side-to-side asymmetry [15]. The normative data of the MSEBT for males recreationally trained (mean ± SD; ANT: 79.2 ± 7.0, PM: 95.6 ± 8.3, PL: 90.4 ± 13.5) [17].

#### Plank test

**Fig. 1:**
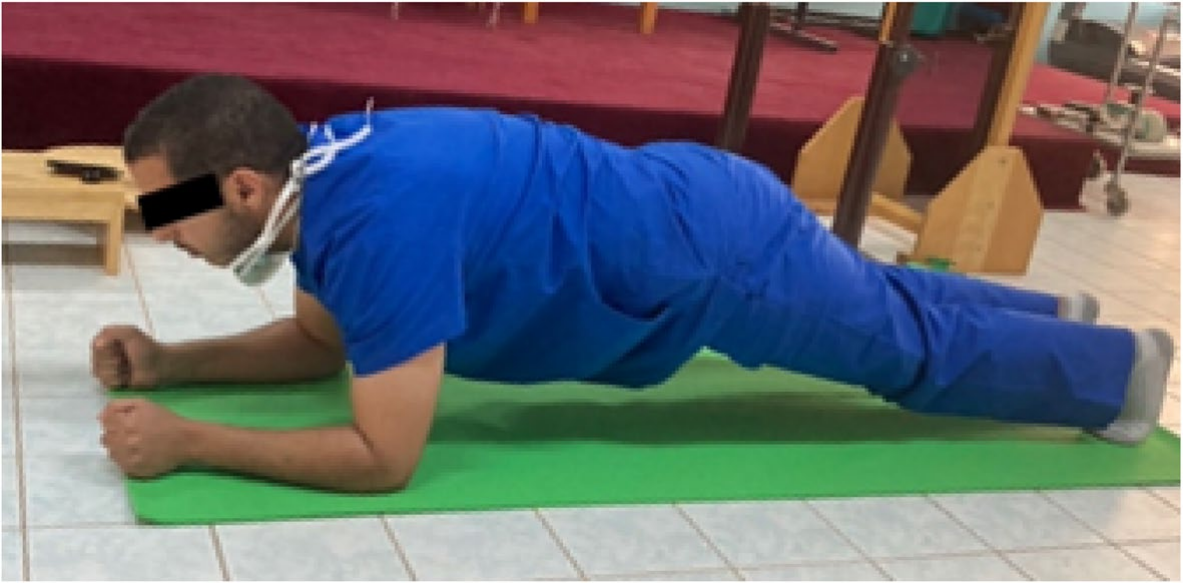
Plank test

The plank exercise test is a position developed to assess the strength of the core stabilization muscles, and it is one of the exercise used to strengthen core stability [18] (Fig. 1). The plank test is reliable on the (ICC = 0.915) and a valid measure to evaluate performance in both older and younger adults [19].

#### Procedures

Before data collection, all subjects read and signed an informed consent document approved by the Ministry of Health’s Institutional Review Board (H-02-J-002) and start to obtain the anthropometric measurements. Participants were explained about the procedure of the tests. Participants were given a practice trial before the actual performance of the test. After the practice trial and before the test begins, each participant was given a few minutes of rest to prepare for the test.

#### Assessment of dynamic balance

To assess the participant’s dynamic balance, MSEBT was performed according to a published protocol (Plisky et al., 2009). Bare-footed participants began the MSEBT with six practice trials in every direction before they underwent the formal testing. Then conducted 3 test trials in each direction and the mean value of the 3 test trials was determined for data analysis. In every trial, the participants were educated to spread foot as far as possible and the opposite foot was maintained in the center. In each reach trial, the greatest reach distance was reported to the nearest 0.5 cm. Reach distances in centimeters (cm) were also standardized to of participant's leg length by dividing the reach distance by the limb length and then multiplying by 100 to allow for the effect of leg length on test results [15].

#### Lower limb girth measurement

Lower limb length is measured by girth tape in the supine position from the anterior superior iliac spine to the medial malleolus. The measure of the leg length using strip measure has proved excellent reliability [15]. The dominant lower limb for the participants was determined based on their answer to the question “which foot do you apply to hit a ball with.”

#### Plank Test

In the Plank test, participants maintain a prone posture in which the forearm bears the weight of the body while the hands make a fist. The elbows are placed shoulder distance apart with the ankle joint at 90° angulation. The abdominal drawing-in maneuver was used to compress the abdomen; the shoulders, trunk hips, and knees were held in a straight line with toes touching the ground (Fig. 1). While performing the plank test exercises, participants were instructed to maintain a neutral pelvis and spine position and breathe regularly. The normative data of the plank test average: 1 to 2 min and the test were ranged from very poor < 15 s to excellent > 6 min (Topendsports, 2021). The test was stopped when (1) the participant became fatigued or voluntarily stopped the test, (2) the participant refused to hold the correct posture, or (3) the participant indicated adverse effects from the test (e.g., headache, dizziness, pain not associated with fatigue).

### Statistical analysis

The independent *t* test was used to compare the mean scores of MSEBT between male administrative and health practitioners. Bivariate correlations (Pearson’s correlation coefficient) were used to measure the linear relationship between the three components of MSEBT and the plank test among participants. The statistical significance amount was set at *p* < 0.05. For data analysis, the SPSS software package (SPSS Inc., Chicago, 2020) was used.

## Results

All data met the assumptions of normality with no outliers. In the male administrative and health practitioner groups, there were no statistically significant differences in age, height, or body mass (Table 1).

**Table 1:** Characteristics of the participants

Variable	Administrative Mean ± SD	Health practitioner Mean ± SD	***p*** value	***t*** test
**Age (years)**	30.86 ± 4.19	33.62 ± 3.78	.085	− 1.793
**Height (cm)**	170.79 ± 7.02	171.54 ± 5.91	.767	− .300
**Mass (kg)**	69.29 ± 10.92	75.69 ± 10.49	.133	− 1.552
**Body mass index ( kg/m**^**2**^**)**	23.77 ± 3.47	25.74 ± 3.62	.159	− 1.453
**Right leg length (cm)**	95.57 ± 5.45	93.46 ± 4.68	.292	1.075
**left leg length (cm)**	95.61 ± 5.44	93.39 ± 4.76	.271	1.126
**Years of Experience**	6.00 ± 2.72	6.15 ± 3.48	.899	− .128
**Hours per week**	31.43 ± 9.03	32.92 ± 9.23	.674	− .425

*cm* centimeters, *kg* kilogram, *kg/m*^*2*^ kilogram per meter square

### Core muscle strength and dynamic balance

The descriptive data of the participants are provided in Table 1. Independent *t* test showed that core muscle strength and dynamic balance revealed a non-significant difference between male administrative and health practitioners (*p* > 0.05) (Table 2).

**Table 2:** Values for MSEBT and plank tests

Test variables	Administrative	Health practitioner	Career difference	95% CI	Mean difference (95% CI)
	Mean ± SD	95% CI	Mean ± SD		
**Anterior (%LL)**					
**Right**	65.7 ± 7.0	62.04–69.3	69.7 ± 4.4	67.3–72.1	− 4.0 (− 8.7 to 0.6)
**Left**	65.3 ± 7.3	61.4–69.2	65.2 ± 18.4	55.2–75.2	0.1 (− 10.8 to 11.1)
**Dominant**	65.5 ± 7.0	61.8–69.2	69.7 ± 4.3	67.4–72.1	− 4.2 (− 8.8 to 0.4)
**Non-dominant**	65.5 ± 7.3	61.6–69.3	70.0 ± 5.9	66.8–73.2	− 4.5 (− 9.8 to 0.7)
**Posteromedial (%LL)**					
**Right**	58.3 ± 12.5	51.7–64.9	66.6 ± 9.4	61.5–71.8	− 8.3 (− 17.2 to 0.5)
**Left**	61.1 ± 11.1	55.3–66.9	66.5 ± 12.4	59.7–73.3	− 5.4 (− 14.7 to 3.9)
**Dominant**	55.4 ± 11.2	49.5–61.3	62.6 ± 9.7	57.3–67.9	− 7.1 (− 15.5 to 1.1)
**Non-dominant**	61.2 ± 11.0	55.5–67.0	66.3 ± 12.5	59.5–73.1	− 5.0 (− 14.3 to 4.3)
**Posterolateral (%LL)**					
**Right**	65.6 ± 11.3	59.7–71.5	69.3 ± 7.8	65.14–73.6	− 3.7 (− 11.5 to 4.0)
**Left**	64.3 ± 12.4	57.8–70.9	70.4 ± 7.8	66.18–74.7	− 6.1 (− 14.4 to 2.2)
**Dominant**	65.2 ± 11.5	59.2–71.3	69.3 ± 8.8	64.59–74.1	− 4.1 (− 12.2 to 4.0)
**Non-dominant**	64.7 ± 12.3	58.2–71.1	70.4 ± 6.7	66.80–74.1	− 5.7 (− 13.7 to 2.2)
**Composite (%LL)**					
**Right**	63.2 ± 9.4	58.3–68.2	68.7 ± 5.1	65.99–71.5	− 5.4 (− 11.5 to 0.5)
**Left**	63.7 ± 9.3	58.8–68.5	69.1 ± 6.9	65.40–72.9	− 5.4 (− 11.98to 1.1)
**Dominant**	63.0 ± 9.5	58.0–68.0	68.8 ± 5.3	65.93–71.7	− 5.7 (− 11.9 to 0.4)
**Non-dominant**	63.9 ± 9.1	59.1–68.7	69.0 ± 6.7	65.43–72.7	− 5.1 (− 11.5 to 1.2)
**Plank test**	63.6 ±3 4.3	45.6–81.6	77.5 ± 26.6	63.0–92.0	− 13.9 (− 38.4 to 10.6)
**ANT difference (cm)**	1.3 ± 1.6	0.49–2.2	1.1± 1.3	0.4–1.87	0.2 (− 1 to 1.4)
**PM difference (cm)**	4.2 ± 2.6	2.87–5.6	3.73± 2.3	2.4–5.03	0.5 (− 1.4 to 2.5)
**PL difference (cm)**	3.6 ± 2.5	2.33–5.0	4.8 ± 3.0	3.1–6.48	− 1.1 (− 3.3 to 1.1)

Note: *MSEBT* modified Star Excursion Balance Test, *LL* lower limb, *SD* standard deviation, *CI* confidence interval

### Anterior leg reach

There was a significant correlation between the plank test with right anterior leg reach (*r* = 0.442, *p* = 0.010), dominance anterior leg reach (*r* = 0.446, *p* = 0.010), and non-dominance anterior leg (*r* = 0.335, *p* = 0.044) and a non-significant correlation with left anterior leg reach distances (*r* = − 0.042, *p* = 0.418). All the other correlations between variables are provided in Tables 3 and 4.

**Table 3:** Correlations between plank test and MSEBT

	***r***	***p*** value	Strength of correlation
**Plank test (s) with anterior reach (%LL) correlation**			
**Right**	.442^a^	.010	Moderate
**Left**	− .042	.418	Weak
**Dominant**	.446^b^	.010	Moderate
**Non-dominant**	.335^a^	.044	Moderate
**Plank test (s) with posteromedial reach (%LL) correlation**			
**Right**	.338^a^	.042	Moderate
**Left**	.275	.083	Weak
**Dominant**	.395^a^	.021	Moderate
**Non-dominant**	.277	.081	Weak
**Plank test (s) with posterolateral reach (%LL) correlation**			
**Right**	.208	.149	Weak
**Left**	.179	.186	Weak
**Dominant**	.211	.145	Weak
**Non-dominant**	.174	.193	Weak
**Plank test (s) with composite score (%LL) correlation**			
**Right**	.355^a^	.035	Moderate
**Left**	.297	.066	Weak
**Dominant**	.355^a^	.034	Moderate
**Non-dominant**	.296	.067	Weak

Note. *s* seconds, *%LL* percentage of lower limb reach, *r* Pearson’s correlation coefficient

^a^Significant at 0.05

^b^Significant at 0.01

**Table 4:** Differences between administrative and practitioner

	***p*** value	***t*** test
**Anterior (%LL)**		
**Right**	.086	− 1.785
**Left**	.981	.024
**Dominant**	.077	− 1.846
**Non-dominant**	.088	− 1.778
**Posteromedial (%LL)**		
**Right**	.065	− 1.934
**Left**	.245	− 1.191
**Dominant**	.089	− 1.769
**Non-dominant**	.278	− 1.109
**Posterolateral (%LL)**		
**Right**	.331	−.991
**Left**	.145	− 1.506
**Dominant**	.313	− 1.031
**Non-dominant**	.149	− 1.489
**Composite (%LL)**		
**Right**	.075	− 1.858
**Left**	.099	− 1.713
**Dominant**	.067	− 1.917
**Non-dominant**	.111	− 1.653
**Plank test (s)**	.254	− 1.168
**ANT difference (cm)**	.731	.348
**PM difference (cm)**	.598	.533
**PL difference (cm)**	.309	− 1.04

*t test* Student’s *t* test, *p* level of significance, *%LL* percentage of lower limb, *cm* centimeters

### Posteromedial leg reach

There was a significant correlation between the plank test with right posteromedial leg reach (*r* = 0.338, *p* = .042), dominance posteromedial leg reach (*r* = 0.395, *p* = 0.021) and non-significant correlation with left posteromedial leg reach (*r* = 0.275, *p* = 0.083), non-dominance posteromedial leg reach (*r* = 0.277, *p* = 0.081).

### Posterolateral leg reach

There was no significant correlation between the plank test with the right, left, dominant, and non-dominant posterolateral leg reach.

### Composite score leg reach

There was a significant correlation between plank test with right composite leg reach (*r* = 0.355, *p* = 0.035), dominance composite leg reach (*r* = 0.355, *p* = 0. 034), and non-significant correlation with left composite leg reach (*r* = 0.297, *p* = 0.066) and non-dominance composite leg reach (*r* = 0.296, *p* = 0.067).

## Discussion

The purpose of this study was to examine the relationship between Plank Test and MSEBT performance among hospital staff. The main findings of our study were that correlations between reach distances in different directions of MSEBT with the plank test were generally weak to moderate. The strongest relationship was found between the MSEBT anterior (ANT) dominant leg reach distances scores with the plank test. And the study also showed a smaller, but statistically significant relationship with ANT right, ANT non-dominant, PM right, PM dominant, right composite score, and dominant composite score. No significant relationship was found between the MSEBT PL leg reach distances scores with the plank test. There was no difference in MSEBT and Plank test scores between administrative and health practitioner workers. Also, no limb differences were found in MSEBT reach performance between administrative and health practitioner workers. This similarity in limbs is consistent with previous literature [20–22] and provides further support that healthy limbs will perform similarly during MSEBT performance.

Unlike previous research on the MSEBT [23–27], the sample in our study was not restricted to a single sport, disability, or gender. This study contained only male hospital employees. The previous study has found no difference in SEBT or MSEBT scores between genders [28, 29]. The literature consistently suggests that trunk muscle exhaustion has a negative impact on coordination. Helbostad et al [30] reported that the balance and functional task performance are impaired with fatigue.

According to Kibler et al., the body generates the requisite rotational torques around the body and produces extremity motion by activating core muscles [9]. In MSEBT, when the subject stands on the stance leg and reaches for the opposite hand, the rectus abdominis muscles and obliques fire before the action to conduct trunk motion, helping the subject to maintain equilibrium. Furthermore, by supplying assistance to the lumbar spine, the multifidi and transverse abdominis muscles will help to maintain dynamic equilibrium during lower extremity movement [31]. According to [32], the core intensity has a certain connection to dynamic equilibrium and may lead to injury prevention.

Different muscles are used in MSEBT performances to reach in different directions. The hamstrings and quadriceps muscles work in both directions. The vastus lateralis is primarily involved in the posteromedial directions of the non-dominant leg's dynamic [33]. The biceps femoris muscle, on the other hand, works in the posterolateral direction. The MSEBT test was performed for both the dominant and non-dominant legs in our sample. In the dominant leg, statistically significant correlations were observed in the ANT and PM directions. Similarly, research conducted among 15 female athletes and 15 nonathletes reported no significant difference in MSEBT after 20 min whole-body fatigue protocol [34]. The authors also reported that the dominant leg reached a maximum test score than the non-dominant leg.

The two screening tests used in our study were relatively easy to implement in clinical settings and also to identify the risk group. The MSEBT took 4–5 min to complete, while the plank test took 2–3 min. For the past few years, Health professionals have experienced work overload and stress due to the COVID-19 pandemic, and these tests will help identify risk groups for preventive measures. Moreover, Most of the existing research has focused on sports personnel, while our study compares the performance of administrative employees with that of health professionals in hospital fieldwork assessed by the same rater.

In line with the hypothesis, a positive correlation between core muscle strength and dynamic balance was found except for MSEBT PL leg reach distances scores with the plank test among male hospital staff with no significant difference between administrative and health practitioners while performing the MSEBT and plank test. On the contrary, a study conducted among 44 lacrosse players reported no correlation between hip external rotators strength with lower extremity balance scores [17]. Different muscles in the lower limb and trunk may contribute uniquely to the scores on MSEBT. The experiment provides new insight into the relationship between the MSEBT of the right dominant leg and with plank test. Overall, it appears that future research is needed to obtain robust evidence about the unique contribution of a different muscle group to the balance and musculoskeletal disorders among health professionals. Identification of muscle groups significantly contributing to the dynamic lower limb balance may help in developing better strategies to encounter health-related problems in health professionals.

## Limitations

There are several limitations to our study. The sample size is small and the generalisability of the study is limited to health care workers in one hospital. There are more objective tests available for the assessment of dynamic balance and core strength such as biodex and force platforms and digital pressure monitors. Therefore, the accuracy of measurement may be flawed. Only the plank test was used in the study to assess core muscle strength however, there are other more comprehensive tests available to measure the timing and quality of core muscle contraction.

## Conclusion

This study reports significant weak to moderate positive correlations between the Plank test of isometric core muscle strength with both the right and dominant of the anterior, posteromedial, and composite score on the MSEBT of the lower limb and also significantly correlation with non-dominant anterior reach. Additionally, no significant difference in core muscle strength or scores on MSEBT was found between the administrative and health practitioner.

## Recommendations

Healthcare workers should consider performing targeted core muscle strengthening exercises for individuals with poor plank tests and MSEBT scores. Furthermore, these conclusions may be important in clinical assumptions when considering the injury prediction abilities of both the weak core muscle and poor dynamic balance. Understanding these relationships will help healthcare professionals improve screening and treatment strategies for workplace illnesses and injuries.

## Data Availability

The datasets used and/or analyzed during the current study are available from the corresponding author on reasonable request.

## Abbreviations

MSEBT: Modified Star Excursion Balance Test
SEBT: Star Excursion Balance Test
M: Mean
SD: Standard deviation
PL: Posteriolateral
PM: Posteriomedial
ANT: Anterior
